# The dynamic interacting landscape of MAPL reveals essential functions for SUMOylation in innate immunity

**DOI:** 10.1038/s41598-017-00151-6

**Published:** 2017-03-07

**Authors:** Karine Doiron, Vanessa Goyon, Etienne Coyaud, Sanjeeva Rajapakse, Brian Raught, Heidi M. McBride

**Affiliations:** 1 Montreal Neurological Institute, McGill University, 3801 University Ave, Montreal, Quebec H3A 2B4 Canada; 2 Princess Margaret Cancer Centre, University Health Network, 101 College St., Toronto, ON M5G 1L7 Canada; 3 Department of Medical Biophysics, University of Toronto, Toronto, Canada

## Abstract

Activation of the innate immune response triggered by dsRNA viruses occurs through the assembly of the Mitochondrial Anti-Viral Signaling (MAVS) complex. Upon recognition of viral dsRNA, the cytosolic receptor RIG-I is activated and recruited to MAVS to activate the immune signaling response. We here demonstrate a strict requirement for a mitochondrial anchored protein ligase, MAPL (also called MUL1) in the signaling events that drive the transcriptional activation of antiviral genes downstream of Sendai virus infection, both *in vivo* and *in vitro*. A biotin environment scan of MAPL interacting polypeptides identified a series of proteins specific to Sendai virus infection; including RIG-I, IFIT1, IFIT2, HERC5 and others. Upon infection, RIG-I is SUMOylated in a MAPL-dependent manner, a conjugation step that is required for its activation. Consistent with this, MAPL was not required for signaling downstream of a constitutively activated form of RIG-I. These data highlight a critical role for MAPL and mitochondrial SUMOylation in the early steps of antiviral signaling.

## Introduction

The first line of host defense against infection is the recognition of invading microbial pathogens or other potential threats by the cell surface Toll-like receptors that sense the presence of bacteria or viruses in the extracellular environment. Within the cell, retinoic acid-inducible gene I (RIG-I) and melanoma differentiation association gene 5 (MDA5) are the two essential innate immune receptors that bind specifically to double-stranded RNA in the cytosol^1^. Upon binding to dsRNA, the helicase domains within RIG-I are opened, thereby exposing the N-terminal caspase recruitment domains (CARD)^2^. This conformational change allows the CARD domains to bind the mitochondrial antiviral signaling protein MAVS (also called CARDIF, VISA and IPS-1^3–6^). MAVS is anchored within both mitochondria and peroxisomes, and upon binding to dsRNA:RIG-I complexes, assembles into a filamentous protein aggregate that recruits the TNF receptor associated factors TRAF2/6, E3 ubiquitin ligases that generate free K63 linked ubiquitin chains^1^. Together this acts as a scaffold to recruit the kinases IKK or TBK1, which phosphorylate IRF3 and MAVS^7^. Phosphorylated IRF3 then dimerizes and translocates to the nucleus to mediate the type I interferon response, acting in concert with the liberation of the transcription factor Nf-κB that drives the transcription of cytokines.

In addition to the multiple roles of ubiquitin and phosphorylation in this signaling cascade, it has also been suggested that the conjugation of SUMO (Small ubiquitin like modifier) is important^8–12^. The conjugation of SUMO to acceptor lysine residues on substrate proteins occurs in a manner analogous to ubiquitination, where an E1 heterodimer first charges the free SUMO and transfers it to an E2 ligase enzyme (Ubc9), which acts in a catalytic manner to conjugate SUMO to an acceptor lysine^13^. There are 3 major SUMO proteins, SUMO1–3, encoded by different genes, and it has been shown that complex SUMO chains can form, as well as mixed chains with ubiquitin^14–17^. The SUMOylation reaction is aided by RING finger containing SUMO E3 ligases, acting as scaffolds to increase the specificity and efficiency of the reaction. Once conjugated to SUMO, the substrate has an altered conformation with various functional consequences including the activation of complex assembly, or disassembly, or stabilization of the protein against ubiquitination^18^. The reaction is rapidly reversed through the action of six distinct SUMO proteases, called Sentrin proteases (SenP)^19^. It has been shown that SenP2 deSUMOylates IRF3, stabilizing the protein against degradation during infection^11^. RIG-I has also been seen as a SUMO substrate, a modification shown to enhance its function^9^. The SUMO E3 ligase for RIG-I was later shown to be the mitochondrial anchored protein ligase MAPL (also called MULAN, MUL1, GIDE, HADES), but in that study the data suggested that SUMOylation played an inhibitory role in antiviral signaling^12^. Therefore, it has been unclear whether SUMOylation plays a positive or negative role in antiviral signaling, which enzymes modulate SUMO conjugation in the antiviral pathways, and the number of substrates that may be modified.

In this study we used mouse embryonic fibroblasts and mice from a germline deletion of MAPL to examine the role of SUMOylation during the host innate response to dsRNA infection. The data reveal an absolute requirement for MAPL in the antiviral response. An unbiased BioID approach identified a number of interacting partners specific to the Sendai infection, including RIG-I. Our data demonstrate a clear role for MAPL and mitochondrial SUMOylation in the activation of RIG-I, a modification essential for the mitochondrial antiviral signaling response.

## Results

**Figure 1:**
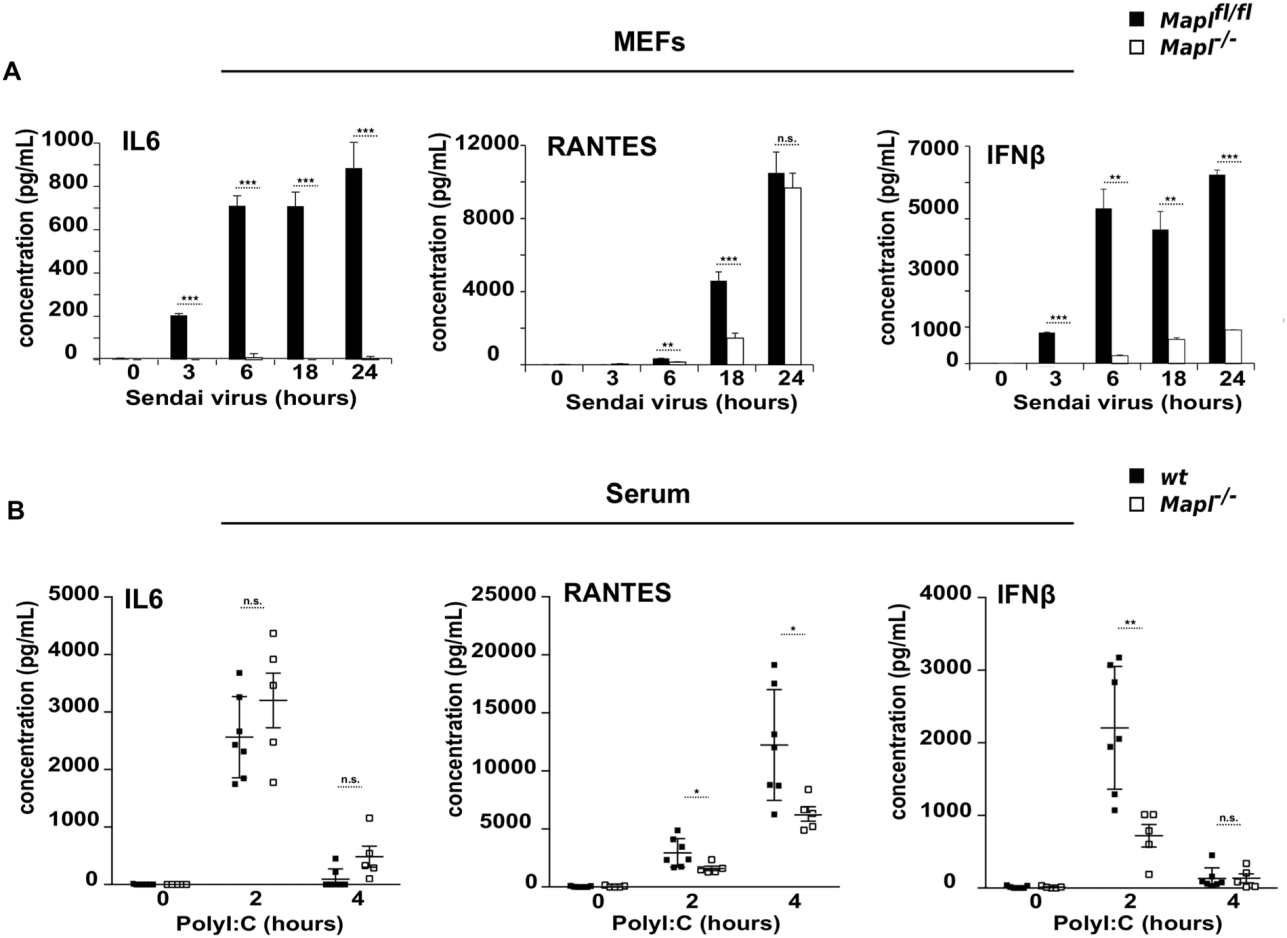
Defective innate immune response *in vitro* and *in vivo* in the absence of MAPL. (**A**) *Mapl* ^*fl/fl*^ and *Mapl* ^−/−^ MEFs were left untreated or infected with Sendai virus (150 HAU/mL) over a time course of 24 hours. IL6, RANTES and IFNβ were measured in supernatants by ELISA. (n = 3). (**B**) Wild-type (WT) (n = 7) and *Mapl* ^−/−^ (n = 5) mice were injected intravenously with saline or PolyI:C for 2 hrs and 4 hrs. Serum IL6, RANTES and IFNβ were measured by ELISA. Results are expressed as the median induction, comparing *Mapl* ^−/−^ to *Mapl* ^*fl/fl*^ or WT. *P < 0.05, **P < 0.01 and ***P < 0.001, n.s., nonsignificant.

In our ongoing efforts to investigate the function of MAPL in mitochondria and peroxisomal membranes we generated a conditional knockout C57/B6 mouse model carrying floxed alleles at exon 2 of the MAPL gene. These mice were crossed with a ubiquitous CMV-Cre strain in order to excise exon 2, ultimately generating germline *Mapl* ^*−/−*^ mice (backcrossed to remove the Cre). These mice are viable, but lean and their complete characterization will be described elsewhere. Given the previous evidence for SUMOylation in innate immune signaling pathways, we generated two sets of embryonic fibroblast cells from wild type and littermate *Mapl* ^*−/−*^ mice, and immortalized them with a retrovirus expressing the E7 gene of type 16 human papilloma virus and a retroviral vector expressing the protein component (hTert) of human telomerase^20^. We infected these cells with Sendai virus and performed ELISA assays to quantify the secretion of IL6, IFNβ and RANTES over a 24-hour time period (Fig. 1A). MEFs lacking MAPL showed almost no IL6 secretion, with a significant reduction in the release of IFNβ or RANTES. The result was confirmed in a second set of MEFs (Supplementary Fig. 1).

To test the requirement for MAPL in an antiviral response in whole animals, we injected PolyI:C, a synthetic analog of double-stranded RNA (dsRNA) and examined cytokine production within the serum at time 0, 2 and 4 hours post-injection (Fig. 1B). Consistent with the data from MEFs, there was a significant reduction in the production of RANTES and IFNβ. However, IL6 secretion was unaffected relative to wild type mice. This may reflect the fact that PolyI:C can also activate TLR3 and signal for IL6 production in a MAVS-independent manner^21^. Nevertheless, there was a clear requirement for MAPL in the efficient activation of the antiviral immune response to PolyI:C.

**Figure 2:**
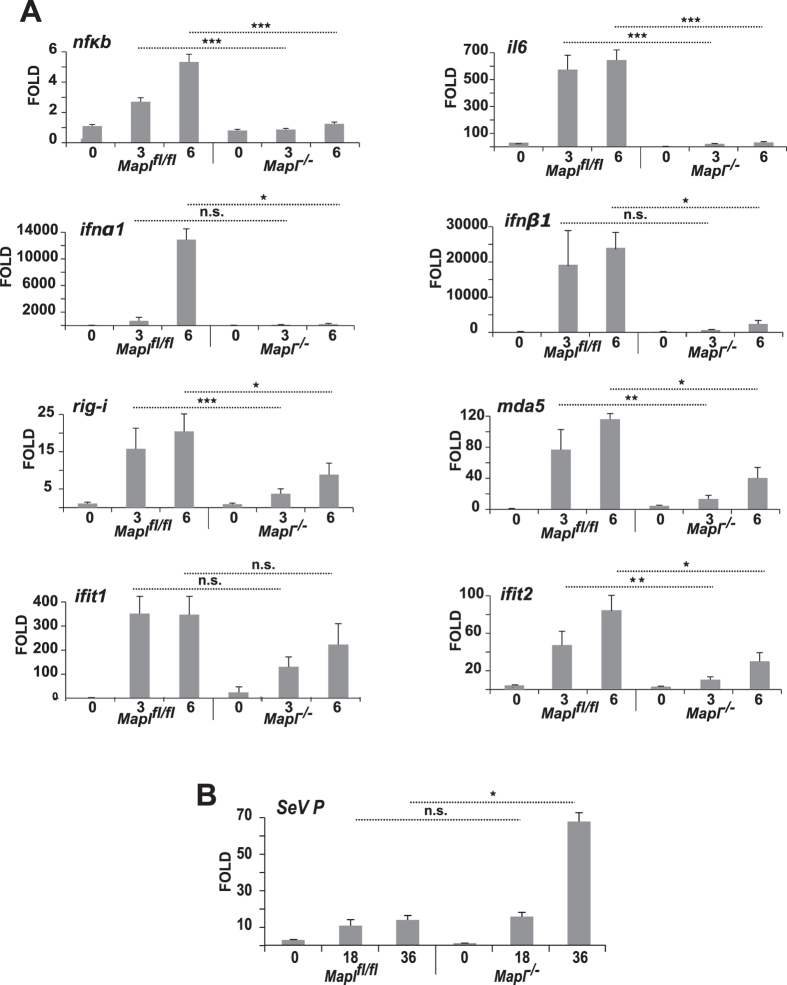
Impaired transcriptional response with the loss of MAPL. (**A**) *Mapl* ^*fl/fl*^ and *Mapl* ^−/−^ MEFs were left untreated or infected with Sendai virus (150 HAU/mL) for 3 or 6 hours, RNA was extracted, and Nf-kB, IL6, IFNα1, IFNβ1, RIG-I, MDA5, IFIT1 and IFIT2 levels were measured by quantitative real-time PCR (n = 3). **(B)** MEFs were left untreated or infected with Sendai virus (150 HAU/mL) for 18 or 36 hours, RNA was extracted, and Sendai virus Protein P level was measured by quantitative real-time PCR (n = 3). Results are expressed as the median induction, comparing *Mapl* ^−/−^ to *Mapl* ^*fl/fl*^. *P < 0.05, **P < 0.01 and ***P < 0.001, n.s., nonsignificant.

The lack of response in *Mapl* ^*−/−*^ MEFs was not due to a block in secretion, rather we observed a upstream block in the transcriptional response, as revealed by qRT-PCR analysis of a number of transcriptionally activated genes including Nf-κB, IL6, IFNα1, IFNβ1, RIG-I, MDA5, IFIT1 (ISG56) and IFIT2 (ISG54) (Fig. 2A)^22^. To further confirm a requirement for MAPL in the transcriptional response, we examined whether *Mapl* ^*−/−*^ cells may show an increase in viral load, since dampening viral translation is an important target of interferon stimulated genes. Indeed, by 36 hours post infection, there was a ~6 fold increase in Sendai Protein P specific mRNA, reflecting the cellular viral load, within MEFs lacking MAPL (Fig. 2B). This increased viral load led to the apoptotic cleavage of PARP by 18 hours within *Mapl* ^*−/−*^ cell (Supplementary Fig. 2). Together, these data demonstrated a requirement for MAPL in the dsRNA innate immune response, likely participating in the early signaling events.

**Figure 3:**
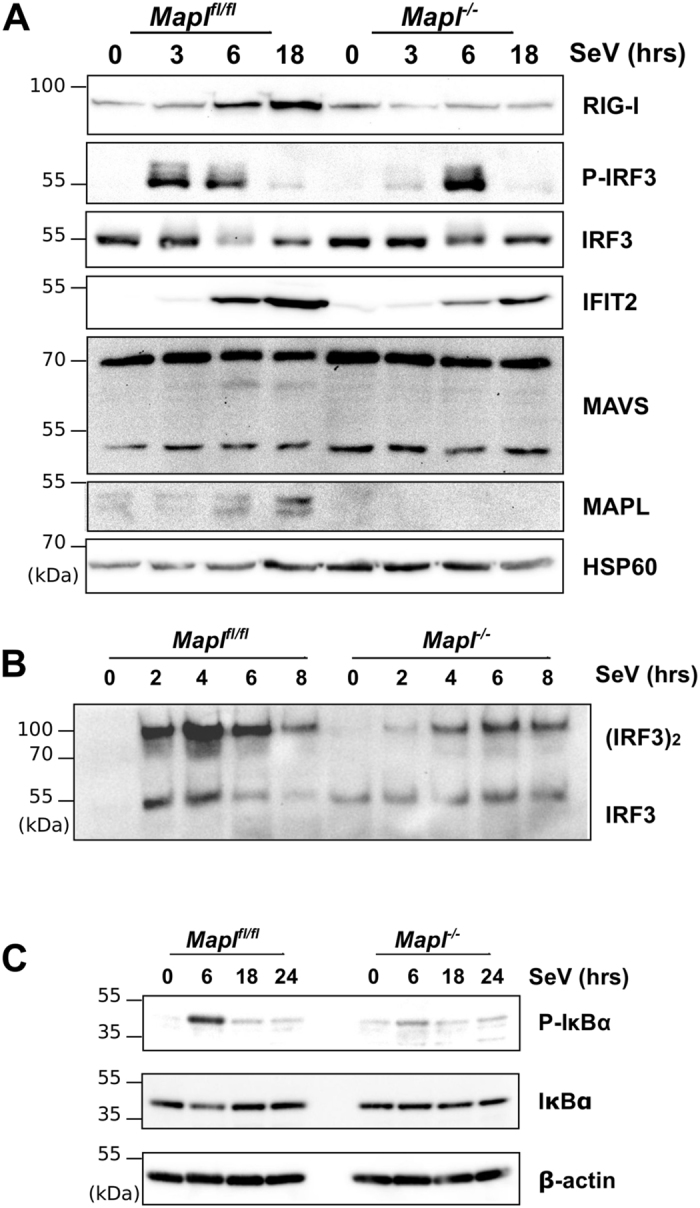
MAPL is required for early steps of immune response to Sendai virus infection. (**A**) *Mapl* ^*fl/fl*^ and *Mapl* ^−/−^ MEFs were left untreated or infected with Sendai virus (150 HAU/mL) for up to 18 hours and lysates were immunoblotted. (**B**) IRF3 dimerization assay. Cells were infected with Sendai virus (150 HAU/mL) for the indicated periods, lysed, ran on a native gel and immunoblotted. (**C**) Nf-κB activation. Cells were left untreated or infected with Sendai virus (150 HAU/mL) for up to 24 hours and lysates were immunoblotted.

Since MAPL is a mitochondrial and peroxisomal membrane protein^23^, we therefore examined the molecular events occurring on the membrane during Sendai virus infection. At 6 hours post infection we observe the expected increase in RIG-I protein levels in the control MEF cells, which did not occur in the *Mapl* ^*−/−*^ cells (Fig. 3A). Consistent with this, there was a delay and inhibition of the phosphorylation/dimerization of IRF3 (Fig. 3A,B). Notably, this was not a complete block, and although we observed a dramatic inhibition in the transcription of antiviral genes (Fig. 2), IRF3 dimerization and phosphorylation was still observed (Fig. 3A,B). Cells lacking MAPL showed a reduction in the phosphorylation of IκBα, leading to less activation of Nf-κB, over 24 hours of infection (Fig. 3C). Consistent with the qRT-PCR data, the protein expression of IFIT2, an interferon stimulated gene involved in the downstream inhibition of viral assembly factors^24, 25^, was also inhibited in cells lacking MAPL.

**Figure 4:**
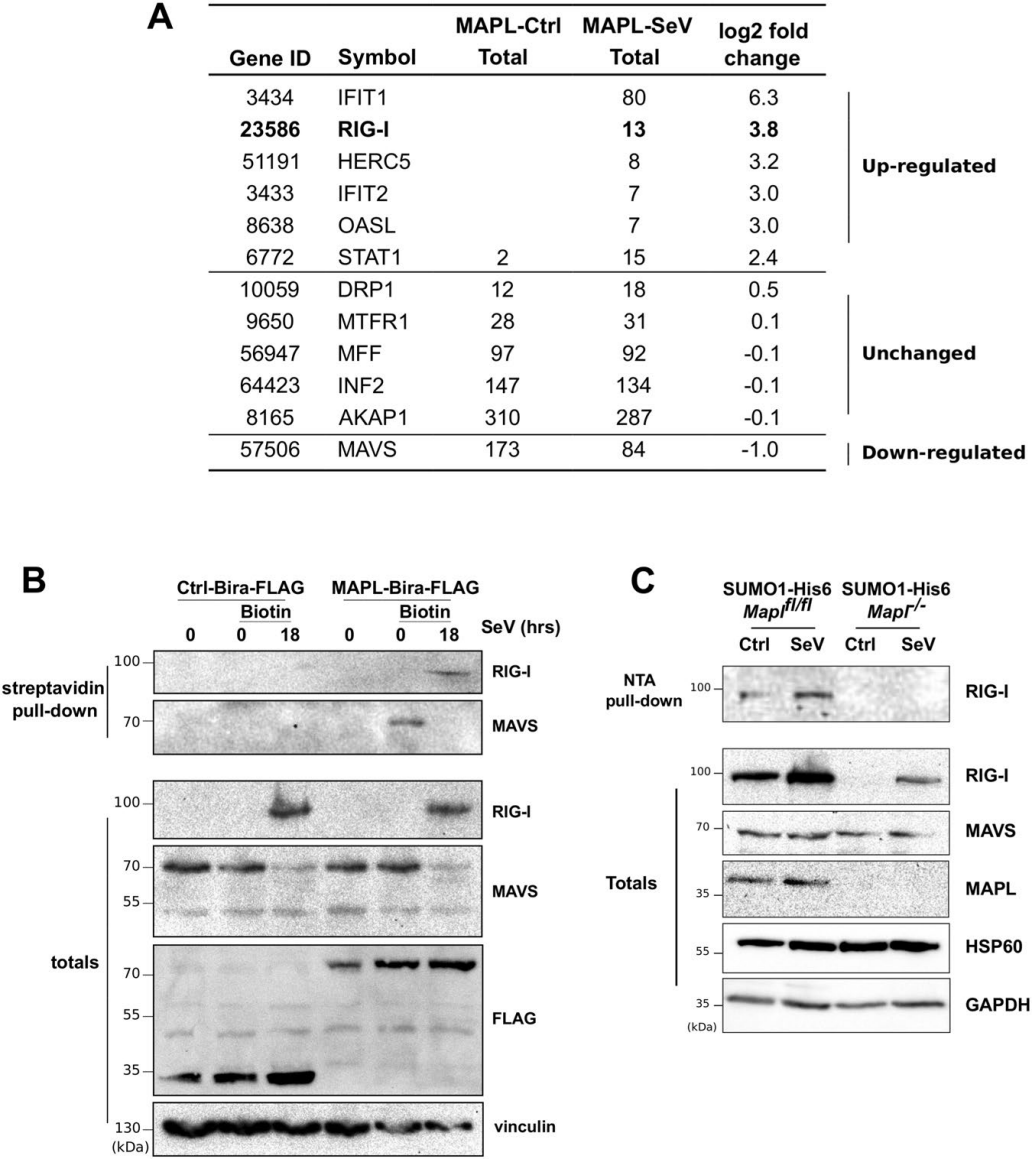
Biotin-labeled interaction landscape identifies RIG-I as a MAPL substrate upon Sendai infection. (**A**) HEK293 cells stably expressing an inducible Tet-ON fusion construct MAPL-BirA-FLAG or Ctrl-BirA-FLAG were induced with tetracycline for 9 hours, and infected (or not) with Sendai virus in the presence of excess biotin within the media for a further 15 hours. Biotinylated proteins were isolated and sequenced by mass spectrometry. Shown are total peptide counts observed for the indicated proteins. Selected proteins showing a greater than 2 fold change in the presence of Sendai virus are shown, along with the top MAPL binding partners related to mitochondrial fission as control. Full dataset is in Supplemental Fig. 2. (**B**) Cells were infected with Sendai virus for 18 hours and tagged proteins were isolated on streptavidin-sepharose and immunoblotted. **(C)** Stable His6-SUMO1 *Mapl* ^*fl/fl*^ and *Mapl* ^−/−^ MEFs were infected with Sendai virus for 18 hours, lysed, and SUMOylated proteins were isolated on Ni-NTA beads and immunoblotted.

To identify direct MAPL binding partners and substrates related to the assembly of this signaling complex, we performed an unbiased protein interactome screen for potential substrates unique to Sendai infected cells. Given that MAPL is a membrane-anchored protein and that MAVS assembles into filamentous complexes, we wished to avoid the use of detergents and centrifugation steps. In addition, as a catalytic enzyme, it can be difficult to trap substrates using simple immunoprecipitation approaches. We therefore turned to an established proximity-dependent biotinylation screening method termed BioID^26, 27^. Briefly, a mutant form of the ~25 kDa *E. coli* BirA biotin ligase (BirA* R118G) was fused to the RING-domain containing C-terminal tail of MAPL^27^. The abortive BirA* mutant protein efficiently activates biotin but is unable to bind the activated product, and thereby releases highly reactive biotinoyl-AMP. Proximal amines (including epsilon amine groups of nearby lysine residues) are thereby covalently labeled with biotin. Numerous studies have now employed this approach to generate high-resolution information on the complex and dynamic protein interactions in cell biological processes, most notably for the events at the centrosome/cilia^28^. In a side-by-side comparison of the same “bait” protein under different conditions, changes in the number of peptides identified for a given interactor implies altered residence time of the interaction throughout the time course of biotin incubation. We generated stable HEK293 Tet-inducible Flp-In cell lines carrying either Flag-BirA or MAPL-Flag-BirA. MAPL expression was induced for 9 hours, and biotin was added to the culture media in control and Sendai infected cells to effect biotinylation of MAPL-proximal proteins over a further 15 hours. Biotinylated proteins were isolated in fully denaturing conditions using streptavidin beads, and identified by mass spectrometry (Fig. 4A). In uninfected cells MAPL was found to interact with expected targets such as Drp1^29, 30^, and a host of mitochondrial fission proteins, including AKAP1^31–33^, INF2^34, 35^, Mff^36–38^, and MFTR1^39^. These data further highlight the established role of MAPL in stabilizing Drp1-mediated ER contacts during division^29^, and validate the BioID approach for studying interactions at the mitochondrial membrane. Although ER/mitochondrial contacts have been implicated in the MAVS signaling pathway^40–42^, the number of peptides identified from the fission machinery were unaltered upon infection (Fig. 4A). However, a number of MAPL proximal interactions were significantly altered upon Sendai infection (Fig. 4A, Supplementary Fig. 3). MAVS was found as a MAPL partner in uninfected cells, and this interaction was reduced during infection. Notably, we also identified a series of biotinylated proteins only in infected cells, including RIG-I and its enhancer, the IFN-inducible oligoadenylate synthetases-like OASL^43^, the interferon stimulated genes IFIT1 and IFIT2^25^, HERC5, an E3 ligase that mediates the conjugation of the ubiquitin-like protein ISG15^44, 45^, and STAT1, a transcription factor responsive to cytokines^46^ (Fig. 4A **, full dataset in** Supplementary Fig. 3).

As we had focused on the signaling complex assembled by oligomerized MAVS, we directly tested potential interactions between MAPL, MAVS and RIG-I. We again induced expression of either Flag-BirA (control) or MAPL-Flag-BirA in cells infected with Sendai virus over an 18-hour period, and isolated biotinylated proteins. Western blot confirmed the interaction with MAVS in uninfected cells, which was lost upon Sendai infection (Fig. 4B). In contrast, RIG-I was biotinylated only in the presence of Sendai virus in a MAPL-dependent manner. Together these data corroborated the mass spectrometry analysis. In order to determine whether MAVS and RIG-I were SUMO substrates of MAPL we stably expressed His6-SUMO1 in control and *Mapl* ^*−/−*^  MEF cells. Cells were infected with Sendai virus for 18 hours and His6-SUMOylated proteins were isolated under high stringency conditions. We observed the MAPL-dependent SUMOylation of RIG-I during Sendai infection, however we could not detect MAVS as a SUMO substrate in either control or infected cells (Fig. 4C, Supplementary Fig. 4A,B). In the absence of MAPL, RIG-I protein levels were not stabilized upon infection, and we could not detect its SUMOylation.

**Figure 5:**
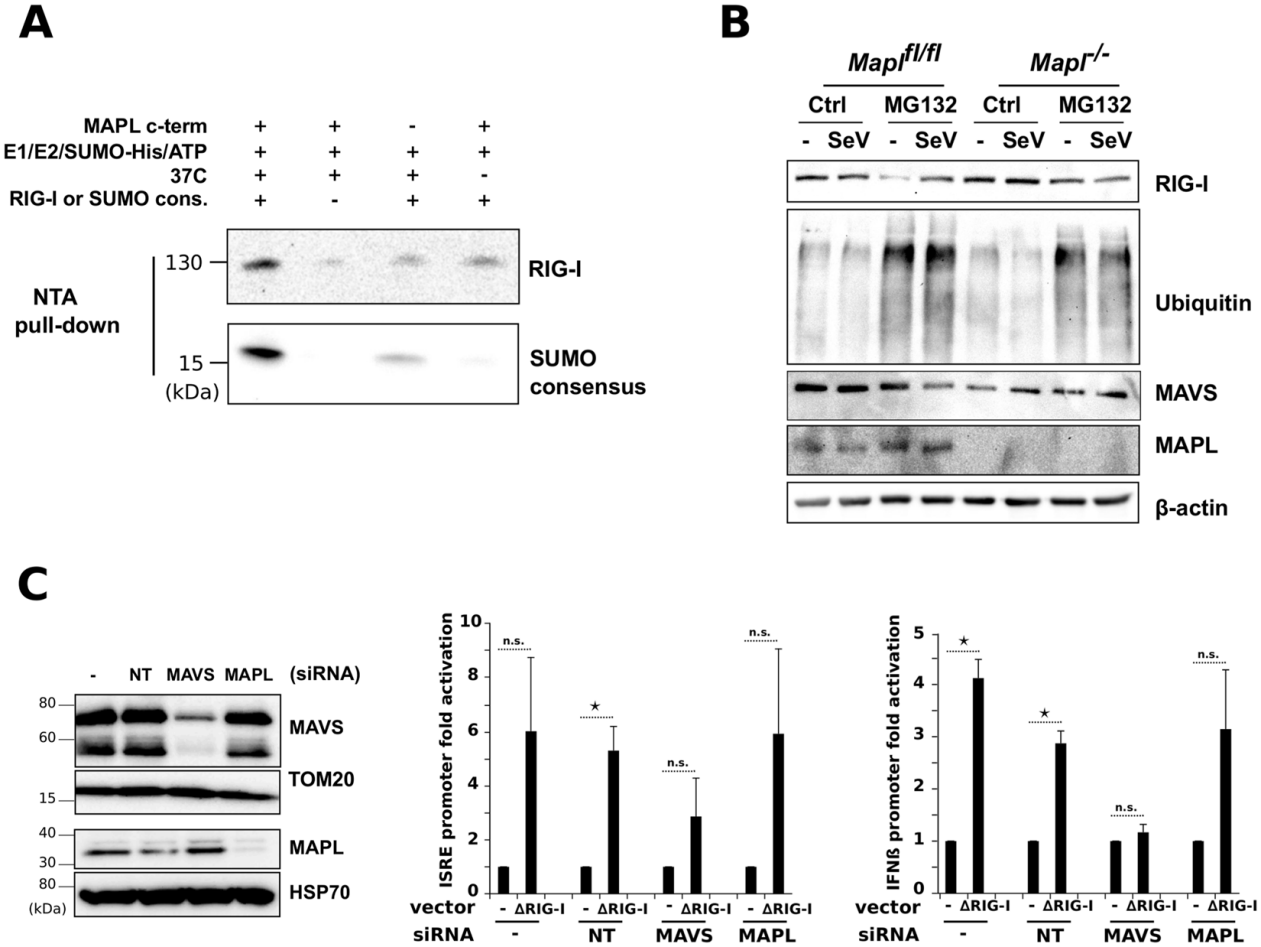
MAPL-dependent SUMOylation of RIG-I is required for activation. (**A**) *In vitro* SUMOylation assay. Recombinant RIG-I protein or SUMO consensus peptide were incubated in the indicated conditions. After the reaction, SUMO-conjugated proteins/peptides were isolated and immunoblotted. (**B**) *Mapl* ^*fl/fl*^ and *Mapl* ^−/−^ MEFs were incubated with MG132 (10 uM) and infected with SeV for 6 hrs then lysates were immunoblotted. (**C**) IFNβ- and ISRE-luciferase reporter assays. U2OS cells were transfected with indicated siRNA for 48 hours then with pIFNβ-luc or pISRE-luc reporter plasmid and pE-CFP (1 μg each) together with 1 μg of myc-ΔRIG-I (constitutively active form of RIG-I). Measures of luciferase activity and CFP were performed 21 hours post transfection. Data represents 2 different experiments done each time in triplicate. Values are reported as SEM ± mean. *P < 0.05, **P < 0.01 and ***P < 0.001, n.s., nonsignificant.

We next tested whether MAPL could SUMOylate recombinant RIG-I in a cell free assay system^30^. We incubated either full-length recombinant RIG-I, or a biotinylated SUMO consensus peptide as a positive control, with His6-SUMO1, SUMO E1 heterodimer, the E2 ligase Ubc9 and the recombinant RING domain of MAPL. Following incubation for 90 minutes in the presence of ATP, SUMOylated proteins or peptides were isolated on NTA-agarose beads. This confirmed that the RING domain of MAPL can SUMOylate both RIG-I and the biotinylated consensus peptide, in an energy and temperature dependent manner. As previously established, we also observed some SUMOylation activity of Ubc9 alone (Fig. 5A, **lane 1 vs. lane 3**), particularly for the conjugation to the peptide containing the SUMO conjugation motif.

Finally, we examined the functional consequences of RIG-I SUMOylation by MAPL. SUMOylation is commonly coupled to ubiquitination events, where SUMO conjugation can stabilize proteins against degradation. To test whether the loss of MAPL destabilized RIG-I we incubated both infected and non-infected cells with MG132 over a 6-hour period to block proteasomal activity (Fig. 5B, Supplementary Fig. 5). In both control and *Mapl* ^*−/−*^ cells, addition of MG132 led to a dramatic accumulation of global ubiquitin conjugates. This pattern was unaffected by Sendai infection. However, the total protein levels of RIG-I and MAVS were unaffected by the loss of MAPL, indicating that the SUMOylation of RIG-I did not interfere with protein stability.

It has been suggested that SUMOylated RIG-I somehow facilitates an interaction with MAVS. This prompted us to test whether a constitutively active form of RIG-I containing only the CARD domains (ΔRIG-I^47^) may override the requirement for MAPL in eliciting the downstream transcriptional response. For this we employed reporter constructs within U2OS cells that drive expression of luciferase by the activation of IFNβ and ISRE, both downstream of MAVS signaling. These cells were silenced for either MAPL or MAVS and transfected with ΔRIG-I (Fig. 5C). In control cells and cells expressing a scrambled siRNA, the transfection of ΔRIG-I led to a robust expression of luciferase driven by both reporter constructs (Fig. 5C). Silencing MAVS led to an ablation of luciferase expression, as previously shown. However the loss of MAPL had no effect on the expression of either reporter driven luciferase in the presence of ΔRIG-I. These data indicate that the requirement for MAPL, and RIG-I SUMOylation is to activate RIG-I, since the constitutively active form shows no impairment in signaling when MAPL is lost.

## Discussion

The activation and assembly of the RIG-I/MAVS signaling pathway is known to require a host of post-translational modifications, including phosphorylation, ubiquitination and SUMOylation^1^. In this study we identify a critical role for MAPL in the activation of RIG-I. Loss of MAPL rendered cells highly susceptible to Sendai viral infection, where the antiviral transcriptional responses were abrogated and viral assembly was enhanced. MAPL has an established role in the SUMOylation of Drp1, stabilizing the oligomeric complex that maintains mitochondrial constrictions at sites of ER contact^29^. This is important to facilitate calcium flux from the ER, and to drive cristae remodeling essential for Bax-dependent apoptosis. The BioID results presented here confirm the direct relationship of MAPL with the fission machinery, however these interactions were unchanged upon Sendai infection. Although our data suggest that MAVS activation does not require sites of SUMO stabilized, Drp1-mediated contacts, this does not exclude additional mechanisms of mitochondrial elongation and ER tethering^40, 48^. Indeed, it has been shown that the induction of mitochondrial hyperfusion alone can activate MAVS, and that this was also dependent upon MAPL (MUL1)^49^.

In this study we identified highly specific MAPL interacting proteins upon Sendai virus infection. First, MAVS was seen to bind MAPL in uninfected cells, and the function of this interaction in steady state is unclear. Importantly, the interaction between MAPL and MAVS is decreased upon recruitment of RIG-I:dsRNA, since we observe a reciprocal interaction between RIG-I and MAVS upon infection. In cells expressing MAPL we demonstrate that RIG-I is SUMOylated in a MAPL-dependent manner during Sendai virus infection, in agreement with the Sendai-specific binding to MAPL, and consistent with a previous study that showed RIG-I SUMOylation during infection^9^. We did not observe any change in RIG-I protein stability in the absence of MAPL, suggesting that this modification is not involved in regulating protein stability or turnover. Instead, we showed that MAPL becomes dispensable for signaling from a constitutively activated form of RIG-I. This indicates a role for SUMOylation in the conformational changes that expose the 2 CARD domains within RIG-I, required to facilitate the assembly of the MAVS signaling complex^50^. A sequence analysis of RIG-I reveals two conserved SUMO consensus sites within RIG-I, one within the helicase domain (mouse K626) and another in the C-terminal RNA binding domain (mouse K889). Conjugation at these sites may aid in the generation of the “open” conformation of RIG-I and/or recognition of the dsRNA. Moreover, these sites lie outside of the CARD domains, consistent with the MAPL-independent activation of MAVS upon transfection of the constitutively active form of RIG-I.

MAPL is not likely a part of the MAVS complex during infection, as the interaction with MAVS is lost at that time. Rather MAPL acts upstream of MAVS complex assembly. This is consistent with the previous evidence that SUMOylated RIG-I showed enhanced ubiquitination and MAVS binding^9^. Interestingly, MAPL is also targeted to peroxisomes in vesicular carriers from the mitochondria^23, 51^. MAVS is also located on peroxisomes, and it has been shown that peroxisomes act as an earlier signaling platform for the antiviral response^52, 53^. We have not dissected the specific contribution of MAPL within mitochondria or peroxisomes in this signaling pathway, but we would speculate that it acts similarly in both locations. In sum our data show that 1) MAPL is required for the innate immune response, 2) that it interacts and SUMOylates RIG-I upon infection, and 3) that MAPL is required for the activation of RIG-I to bind MAVS and signal the transcriptional response.

Lastly, the BioID identified a series of unexpected MAPL targets that act much later in the antiviral response, particularly in the inhibition of viral assembly. IFIT1 and IFIT2 play roles in binding viral mRNA and interfering with the translation of viral proteins^25^. Indeed, IFIT1 was the strongest Sendai-specific hit in the BioID. It has been suggested that mitochondria may surround sites of viral replication^48^, and our data hint towards a role for MAPL in the downstream events in the immune response as well. However, the loss of MAPL blocked the antiviral immune response at the level of RIG-I, which makes it challenging to evaluate the functional requirements for MAPL in downstream processes. Future work will focus on the contribution of SUMOylation in the antiviral activities of the additional interacting proteins identified with the BioID approach. For now we have set the stage for a systematic analysis of the interaction landscape that will provide important insights into the dynamic events that accompany the antiviral immune response.

## Methods

All lab protocols were approved by the Biohazards Committee, at the Montreal Neurological Institute, with federal licensing acquired for the use of Sendai virus. All animal protocols were carried out following the approval of the Montreal Neurological Institute Animal Care Committee, in accordance with the Canadian Council on Animal Care (CCAC), the University Animal Care Committee (UACC), and the MNI Animal Care and Use Program guidelines and policies and all other applicable requirements.

### Reagents

ELISA kits for IL6 (DY406) and RANTES (DY478) were from R&D systems and Verikine IFNβ (42400-1) from PBL; anti-MAPL (HPA017681), anti-β-actin (A2228), anti-FLAG (A8592) and anti-vinculin (V4505) from Sigma, anti-RIG-I (3743), anti-phospho-IRF3 (4947), anti-IRF3 (4302), anti-MAVS rodent specific (4983), anti-IκBα (4812), anti-phospho-IκBα (2859), anti-GST (2624), anti-Ubiquitin (3936) from Cell Signaling, anti-MAVS human (AB1871) from Enzo lifesciences, anti-IFIT2 (NBP2-15180) from Novus Biologicals, anti-Hsp60 (sc-136291) from Santa Cruz, anti-biotin (200-002-211) from Jackson ImmunoResearch, streptavidin-HRP (N100) from ThermoFisher Scientific; Sendai virus Cantell strain from Charles River; recombinant RIG-I from Novus Biologicals Canada; PolyI:C from Invivogen and MG132 (C2211) from Sigma.

### Primary skin fibroblast isolation and immortalization

To isolate mouse embryonic fibroblasts (MEFs), *Mapl* ^*fl/fl*^ or *Mapl* ^−/−^ embryos (as littermate controls) were collected in sterile ice-cold PBS at embryonic day E13-E14. Tissue from the skin were collected and digested in 500 μL DMEM containing trypsin (Sigma). Trypsinization was stopped by addition of 7 ml of DMEM containing 0.57 mg/ml trypsin inhibitors (Roche) and 0.7 mg/ml DNAse I (Roche). Samples were centrifuged at 5 min at 3000 rpm at room temperature and pellets were resuspended in 500 μL of the same solution and centrifuged again using the same conditions. Pellet was resuspended in 1 ml of complete media (DMEM supplemented with 4 mM glutamine) and plated on a 10-cm dish. Fibroblasts were immortalized with a retrovirus expressing the E7 gene of type 16 human papilloma virus and a retroviral vector expressing the protein component (hTert) of human telomerase^20^. Fibroblasts were cultured in Dulbecco's modified Eagle medium (DMEM) supplemented with 10% fetal calf serum.

### Cytokine/chemokine induction

*Mapl* ^*fl/fl*^ and *Mapl* ^−/−^ MEFs were plated in 96 well plates and left untreated or infected with Sendai virus (150 HAU/mL). Supernatant was collected at each time point (0, 3, 6, 18 and 24 hrs, in triplicate) and ELISA was performed to measure the concentration of each cytokine/chemokine. Experiment was repeated three times (n = 3).

### *In vivo* activation of antiviral signaling

Wild-type (n = 7) or *Mapl* ^−/−^ mice (n = 5) were injected intravenously with 200 μg PolyI:C LMW (Invivogen) or saline. Blood was collected prior to injection (time 0), then 2 hours and 4 hours post-injection. Serum levels of IL6, RANTES and IFNβ were measured by ELISA.

### IRF3 dimerization assay

Cells were lysed in native buffer (20 mM Tris-HCl pH 7.5, 150 mM NaCl, 10% Glycerol, 0.5% NP-40, 1 mM sodium orthovanadate and 0.5% sodium deoxycholate) supplemented with protease inhibitor cocktail. 25 ug proteins were ran on 9% native gel, transferred to nitrocellulose and immunoblotted with antibody against mouse IRF3.

### RNA isolation and qRT-PCR

Total RNA from *Mapl* ^*fl/fl*^ and *Mapl* ^−/−^ untreated or infected with 150 HAU/mL SeV for different time periods (in triplicate) were prepared using RNeasy Plus (QIAgen). Total RNA was treated with DNAse (New England Biolabs), then reverse transcribed with random primers using the High Capacity sDNA Reverse Transcription Kit (Life Technologies) as described by the manufacturer. Before use, RT samples were diluted 1:5. Gene expression was determined using assays designed with the Universal Probe Library (UPL) from Roche (www.universalprobelibrary.com). For each qPCR assay, a standard curve was performed to ensure the efficacy of the assay is between 90% and 110%. qPCR reactions were performed using 5–25 ng of cDNA samples, the TaqMan Advanced Fast Universal PCR Master Mix (Life Technologies), 2 µM of each primer. Primer sequences used are as follows: *Rig-I*, reverse 5′-gcagaactggaacaggtcgt-3′ and forward 5′-tgttcgaagtccgggatg-3′; *Ifna1*, reverse 5′-acccagcagatcctgaacat-3′ and forward 5′-aatgagtctaggagggttgtattcc-3′; *Mda5*, reverse 5′-ctattaaccgtgttcaaaacatgaa-3′ and forward 5′-ggatactttgcacctgcaattc-3′; *Ifit1*, reverse 5′-tctaaacagggccttgcag-3′ and forward 5′-gcagagccctttttgataatgt-3′; *Ifit2*, reverse 5′-caatgcttaggggaagctga-3′ and forward 5′-tgatttctacttggtcaggatgc-3′; *Il6*, reverse 5′-gctaccaaactggatataatcagga-3′ and forward 5′-ccaggtagctatggtactccagaa-3′; *Ifnb1*, reverse 5′-ctggcttccatcatgaacaa-3′ and forward 5′-agagggctgtggtggagaa-3′; *Nfkb1*, reverse 5′-cactgctcaggtccactgtc-3′ and forward 5′-ctgtcactatcccggagttca-3′; *SeV PP protein*, reverse 5′-tgttatcggattcctcgacgcagtc-3′ and forward 5′-tactctcctcacctgatcgattatc-3′. The Viia7 qPCR instrument (Life Technologies) was used to detect the amplification level and was programmed with an initial step of 3 minutes at 95 °C, followed by 40 cycles of: 5 sec at 95 °C and 30 sec at 60 °C. All reactions were run in triplicate and the average values of Ct were used for quantification. The relative quantification of target genes was determined using the ΔΔCT method. Briefly, the Ct (threshold cycle) values of target genes were normalized to an endogenous control gene (ΔCT = Ct_target_ − Ct_CTRL_) and compared with a calibrator: ΔΔCT = ΔCt_Sample_ − ΔCt_Calibrator_. Relative expression (RQ) was calculated using the Sequence Detection System (SDS) 2.2.2 software (Applied Biosystems) and the formula is RQ = 2^−ΔΔCT^.

### BioID

BioID was carried out essentially as described previously^54^. In brief, the full-length human MAPL (BC014010) coding sequence was amplified by PCR and cloned into a pcDNA5 FRT/TO BirA-FLAG expression vector (MAPL-AscI_Fwd: tataGGCGCGCCaATGGAGAGCGGAGGGCGGCCCTCG; MAPL-NotI_Rev: ttaaGCGGCCGCGCTGTTGTACAGGGGTATCACCCG). Using the Flp-In system (Invitrogen), 293 T-REx Flp-In cells stably expressing MAPL-BirA-Flag were generated. After selection (DMEM + 10% FBS + 200 μg/ml hygromycin B), 10 × 150 cm^2^ plates of subconfluent (60%) cells were incubated for 9 hrs in complete media supplemented with 1 μg/ml tetracycline, then 50 μM biotin was added and cells were untreated or infected with Sendai virus for 15 hours, for a total of 24 hours. Cells were collected and pelleted (2000 rpm, 3 min), the pellet was washed twice with PBS, and dried pellets were snap frozen. Pellets were lysed in 10 ml of modified RIPA lysis buffer (50 mM Tris-HCl, 150 mM NaCl, 1 mM EDTA, 1 mM EGTA, 1% Triton X-100, 0.1% SDS, 1:500 protease inhibitor cocktail, 250 U Turbonuclease, pH 7.5) at 4C for 1 hr, then sonicated to disrupt visible aggregates. The lysates were centrifuged at 35,000 g for 30 min. Clarified supernatants were incubated with 30 μl packed, pre-equilibrated Streptavidin-sepharose beads at 4C for 3 hr. Beads were collected by centrifugation, washed 6 times with 50 mM ammonium bicarbonate pH 8.3, and treated with TPCK-trypsin (16 hr at 37C). The supernatant containing the tryptic peptides was collected and lyophilized. Peptides were resuspended in 0.1% formic acid and 1/6^th^ of the sample was analyzed per MS run.

High performance liquid chromatography was conducted using a pre-column (Acclaim PepMap 50 mm × 100 um inner diameter pre-column) and Acclaim PepMap (500 mm × 75 um diameter; C18; 2 um;100 Å) RSLC (Rapid Separation Liquid Chromatography) column (Thermo Fisher Scientific, Waltham, MA), running a 120 min reversed-phase buffer gradient at 250 nl/min on a Proxeon EASY-nLC 1000 pump in-line with a Thermo Q-Exactive HF quadrupole-Orbitrap mass spectrometer. A parent ion scan was performed using a resolving power of 60,000, then up to the twenty most intense peaks were selected for MS/MS (minimum ion count of 1000 for activation), using higher energy collision induced dissociation (HCD) fragmentation. Dynamic exclusion was activated such that MS/MS of the same *m/z* (within a range of 10 ppm; exclusion list size = 500) detected twice within 5 s was excluded from analysis for 15 s. For protein identification, Thermo. RAW files were converted to. mzXML format using Proteowizard^55^ then searched using X!Tandem^56^ against the human Human RefSeq Version 45 database (containing 36,113 entries). Search parameters specified a parent ion mass tolerance of 10 ppm, and a MS/MS fragment ion tolerance of 0.4 Da, with up to 2 missed cleavages allowed for trypsin. Variable modification +16@M and W, +32@M and W, +42@N-terminus, +1@N and Q were allowed. Proteins identified with a ProteinProphet cut-off of 0.85 (corresponding to ≤1% FDR) were analyzed with SAINT Express v.3.3. Sixteen control runs were used for comparative purposes, comprising 8 runs of BioID conducted on untransfected 293 T-REx cells and 8 runs of BioID conducted on 293 T-REx cells expressing FlagBirA* only.

### Ni-NTA pulldown

Stable SUMO1-His6-*Mapl* ^*fl/fl*^ and SUMO1-His6-*Mapl* ^−/−^ were left untreated or infected with Sendai virus (150 HAU/mL) for 18 hours. Cells were lysed in Guanidine lysis buffer (6 M Guanidine, 0.1 M NaH2PO4, 10 mM imidazole, 10 mM Tris-HCl pH 8.0, 20 mM 2-chloroacetamide and 10 mM b-mercaptoethanol) and sonicated. 1 mg of proteins were incubated with 50 uL of pre-equilibrated Ni-NTA beads at 4C for 4 hours. Pellets were washed once with 6 M Guanidine buffer then with 8 M Urea. Precipitated proteins were eluted by boiling in Laemmli buffer, ran on SDS-PAGE and immunoblotted using antibodies to mouse RIG-I, MAVS and vinculin as a control.

### SUMOylation assay

The SUMO conjugation assay was performed using 50 nM SUMO E1, 250 nM Ubc9, 10 uM His6-SUMO1, 20 uM of a consensus biotinylated peptide, an ATP-regenerating system (2.5 U creatine kinase, 125 nM creatine phosphate, 5 mM ATP) and 200 ng of recombinant RIG-I. The conjugation reactions were incubated at 30 °C for 90 minutes. Ni-NTA beads were washed in 6 M Guanidine buffer, then incubated with the conjugation reactions overnight at 4 °C, and finally washed once in Guanidine buffer then with 8 M Urea. Precipitated proteins were eluted by boiling in Laemmli buffer, ran on SDS-PAGE and immunoblotted using antibodies to mouse RIG-I, GST and streptavidin-HRP (biotinylated SUMO consensus).

### IFNβ- and ISRE-luciferase reporter assays

U2OS cells were transfected with siRNA for NT (non-targeting), MAVS or MAPL for 48 hours, then with pIFNβ-luc or pISRE-luc reporter plasmid and pE-CFP (1 μg each, a kind gift from Dr. Rongtuan Lin, Lady Davis Institute, McGill University) together with 1 μg myc-ΔRIG-I (constitutively active form of RIG-I, also a gift from Rongtuan Lin, McGill University). Conditions were done in triplicates each time (n = 2). Measures of luciferase activity were performed 21 hours after transfection.

### Statistical analysis

All values are expressed as mean ± SD. Unless stated otherwise statistical significance was tested by the unpaired two-tailed Student's *t* test. *P* values less than 0.05 were considered to represent statistically significant differences.

## Electronic supplementary material

Supplementary Information
