# Supplementary material for: The dynamic interacting landscape of MAPL reveals essential functions for SUMOylation in innate immunity
Source: Sci Rep. 2017 Mar 7;7:107. doi: 10.1038/s41598-017-00151-6 (PMC5427825; doi:10.1038/s41598-017-00151-6)

Supplementary Information

The dynamic interacting landscape of MAPL reveals essential functions for SUMOylation in innate immunity.

Karine Doiron[1], Vanessa Goyon[1], Etienne Coyaud[2], Sanjeeva Rajapakse[1], Brian Raught[2,3] and Heidi M. McBride[1*]

[1]Montreal Neurological Institute, McGill University, 3801 University Ave, Montreal, Quebec H3A 2B4.

[2]Princess Margaret Cancer Centre, University Health Network, 101 College St., Toronto, ON M5G 1L7 Canada

[3]Department of Medical Biophysics, University of Toronto

*Corresponding author

Tel: 514 398 1808

Email: Heidi.mcbride@mcgill.ca

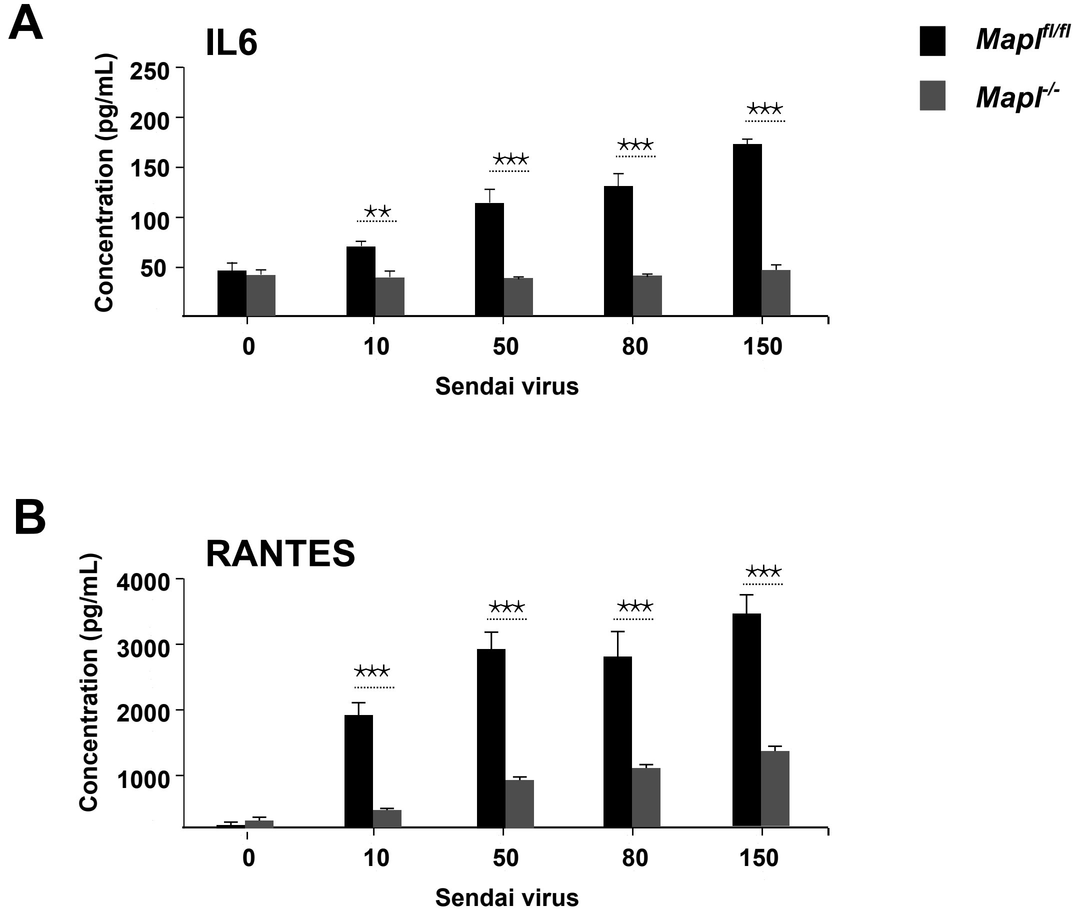

Figure S1. MEFs derived from a second MAPL$^{-/-}$ mouse confirm inhibition of IL6 and RANTES secretion by ELISA. MEFs were left untreated of infected with Sendai virus at indicated MOI for 18 hours. IL6 and RANTES were measured in supernatants by ELISA (n=3).

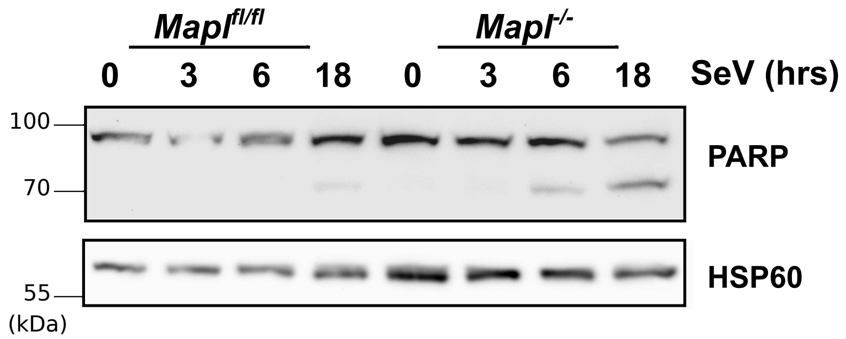

Figure S2. Earlier PARP cleavage in Mapl$^{-/-}$ following SeV infection. MEFs were left untreated or infected with Sendai virus (150 HAU/mL) for up to 18 hours and lysates were immunoblotted.

Gene ID	Symbol	MAPL-Ctrl Total	MAPL-SeV Total	log2 fold change	
3434	IFIT1		80	6.3	
55967	NDUFA12		13	3.8	
**23586**	**RIG-I**		**13**	**3.8**	
85461	TANC1		11	3.6	
51191	HERC5		8	3.2	
6945	MLX		8	3.2	
9100	USP10		7	3.0	
56267	CCBL2		7	3.0	
3433	IFIT2		7	3.0	
118881	COMTD1		7	3.0	**Up-regulated**
8638	OASL		7	3.0	
5879	RAC1		6	2.8	
10558	SPTLC1		6	2.8	
4191	MDH2		6	2.8	
54471	SMCR7L		6	2.8	
3157	HMGCS1		6	2.8	
5194	PEX13		5	2.6	
6905	TBCE		5	2.6	
10802	SEC24A		5	2.6	
6772	STAT1	2	15	2.4	
10059	DRP1	12	18	0.5	
9650	MTFR1	28	31	0.1	
56947	MFF	97	92	-0.1	**Unchanged**
64423	INF2	147	134	-0.1	
8165	AKAP1	310	287	-0.1	
57506	MAVS	173	84	-1.0	**Down-regulated**

Figure S3. Extended BioID of MAPL landscape upon Sendai virus infection. HEK293 cells stably expressing an inducible Tet-ON fusion construct MAPL-BirA-FLAG or Ctrl-BirA-FLAG were induced with tetracycline for 9 hours, and infected (or not) with Sendai virus in the presence of excess biotin within the media for a further 15 hours. Biotinylated proteins were isolated and sequenced by mass spectrometry. Shown are total peptide counts observed for the indicated proteins. Proteins showing a greater than 2 fold change in the presence of Sendai virus are shown, along with the top MAPL binding partners related to mitochondrial fission as control.

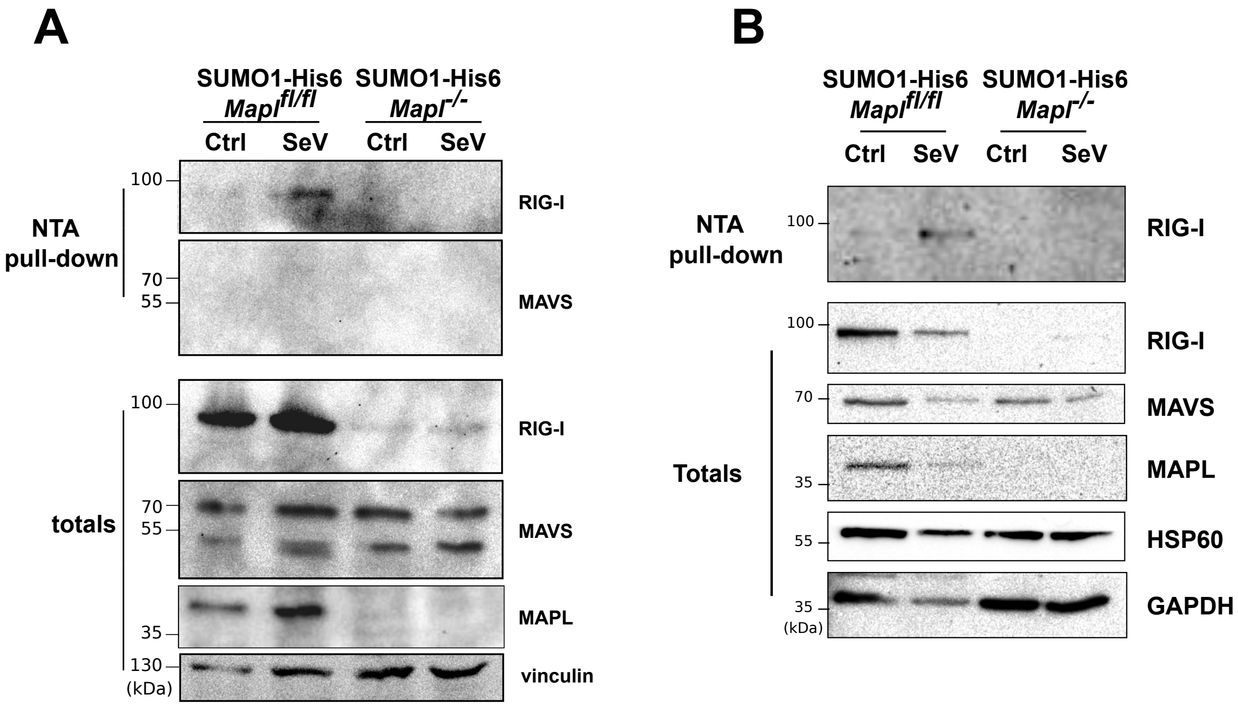

Figure S4. (A, B) Replicate experiments from Figure 4C. SUMO1 stable *Mapl*$^{fl/fl}$and *Mapl*$^{-/-}$ were infected with Sendai virus for 18 hrs, lysed, and SUMOylated proteins were isolated with Ni-NTA beads and immunoblotted.

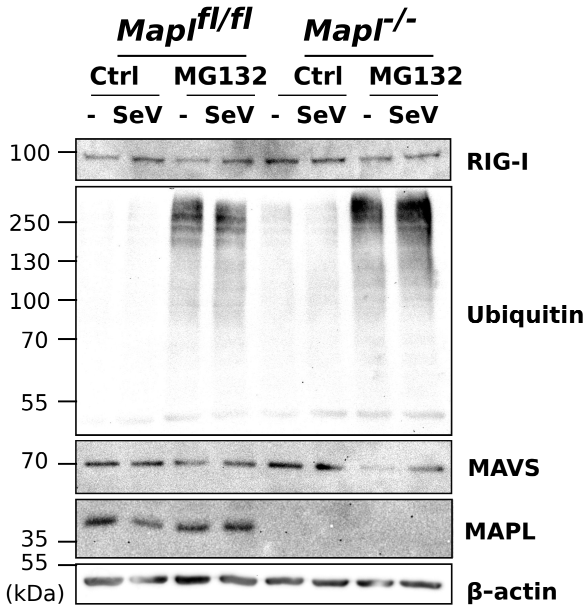

Figure S5. Replicate experiment from Figure 5B. *Mapl*$^{fl/fl}$ and *Mapl*$^{-/-}$ MEFs were incubated with MG132 and infected with Sendai virus for 6 hrs and then lysates were immunoblotted.

Figure 3A

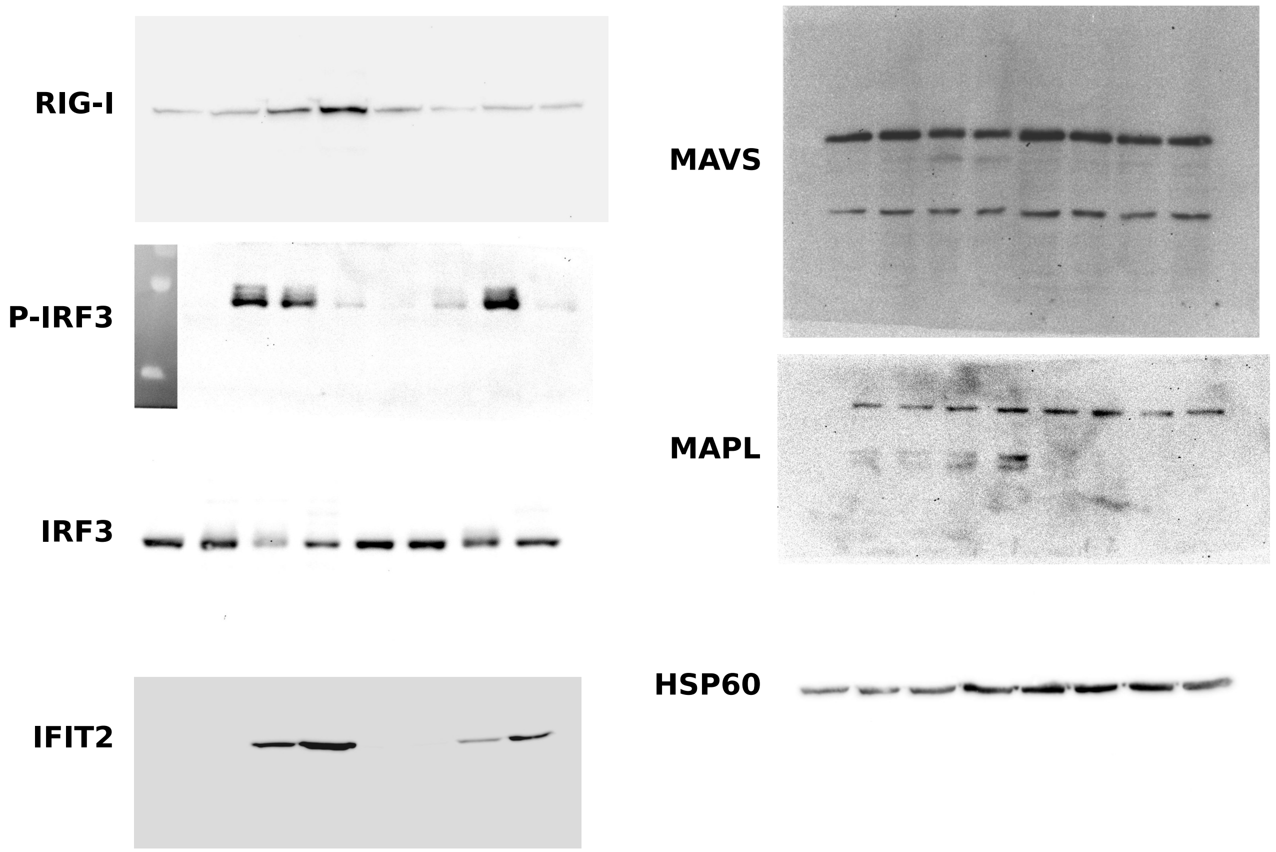

Figure 3C

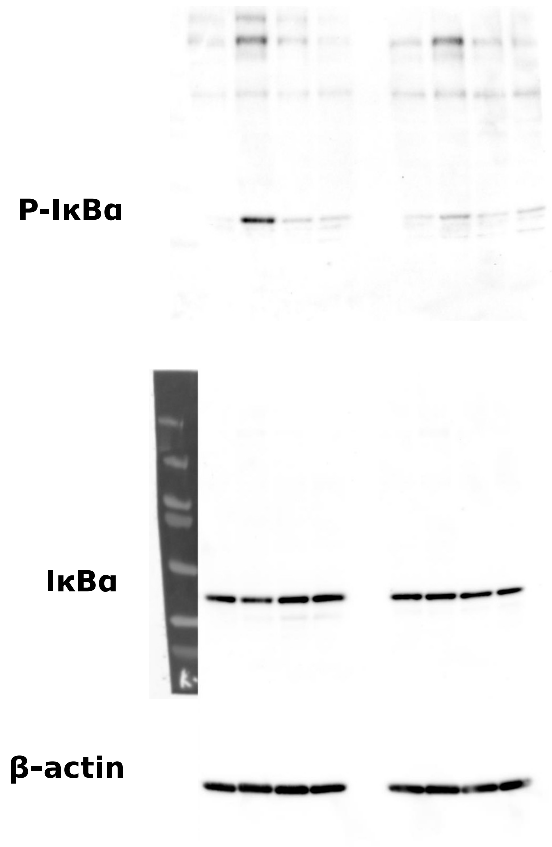

Figure 3B

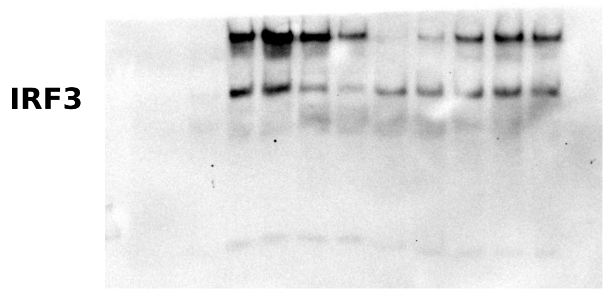

Figure 4B

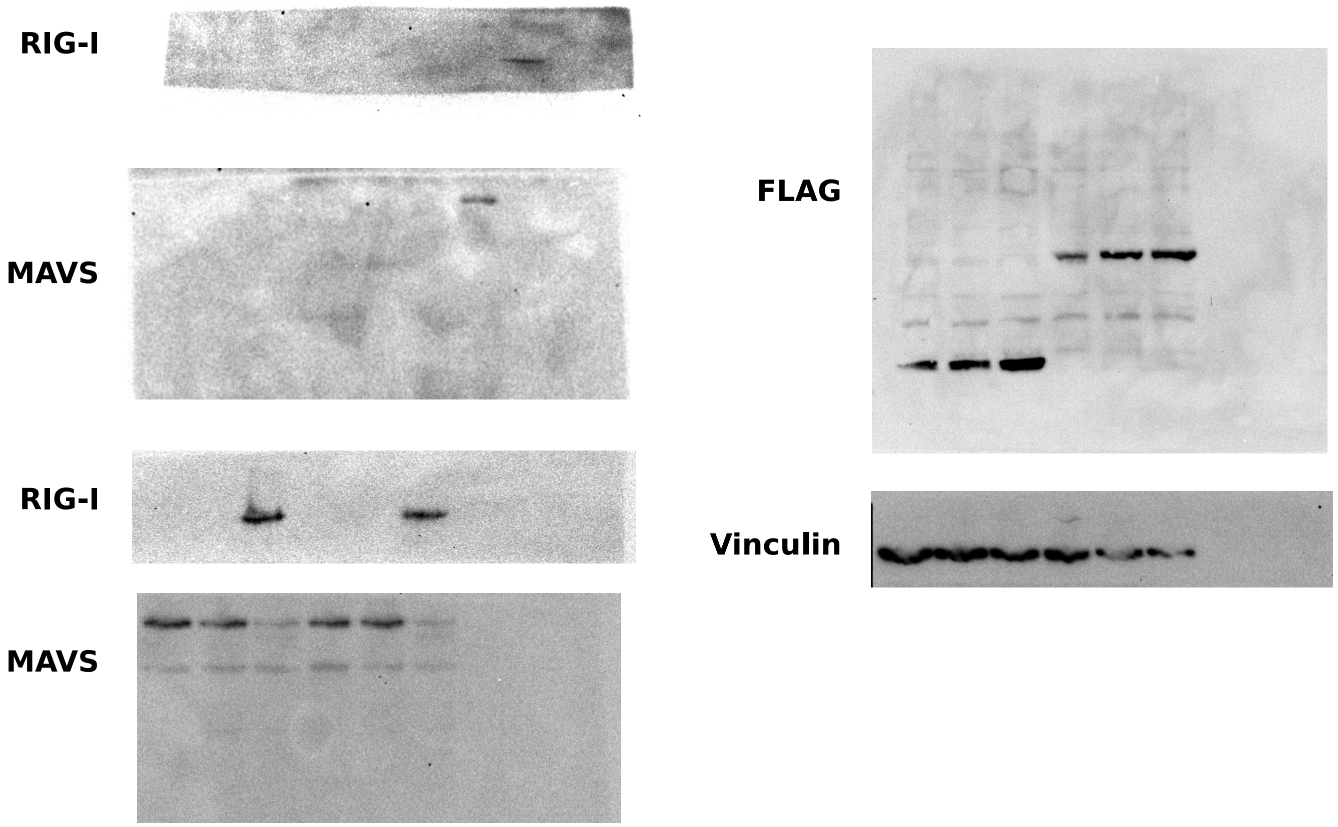

Figure 4C

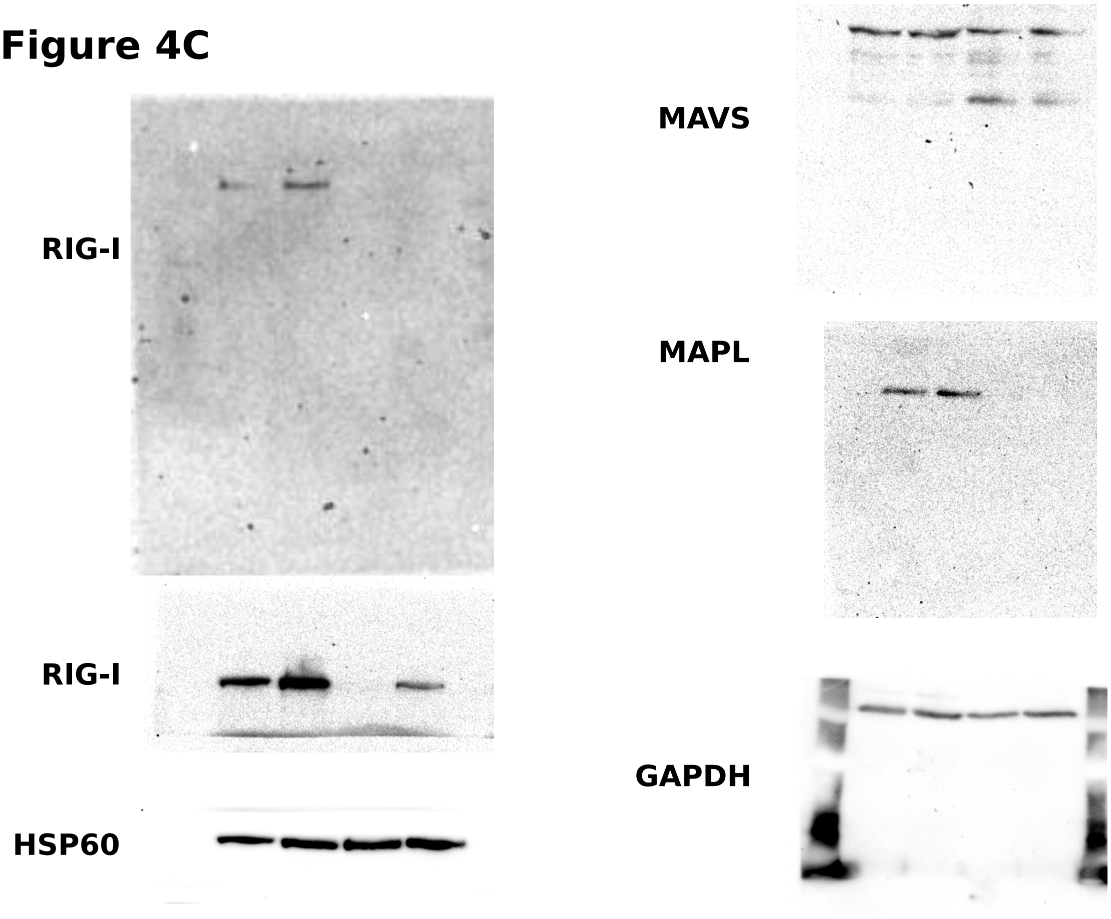

Figure 5A

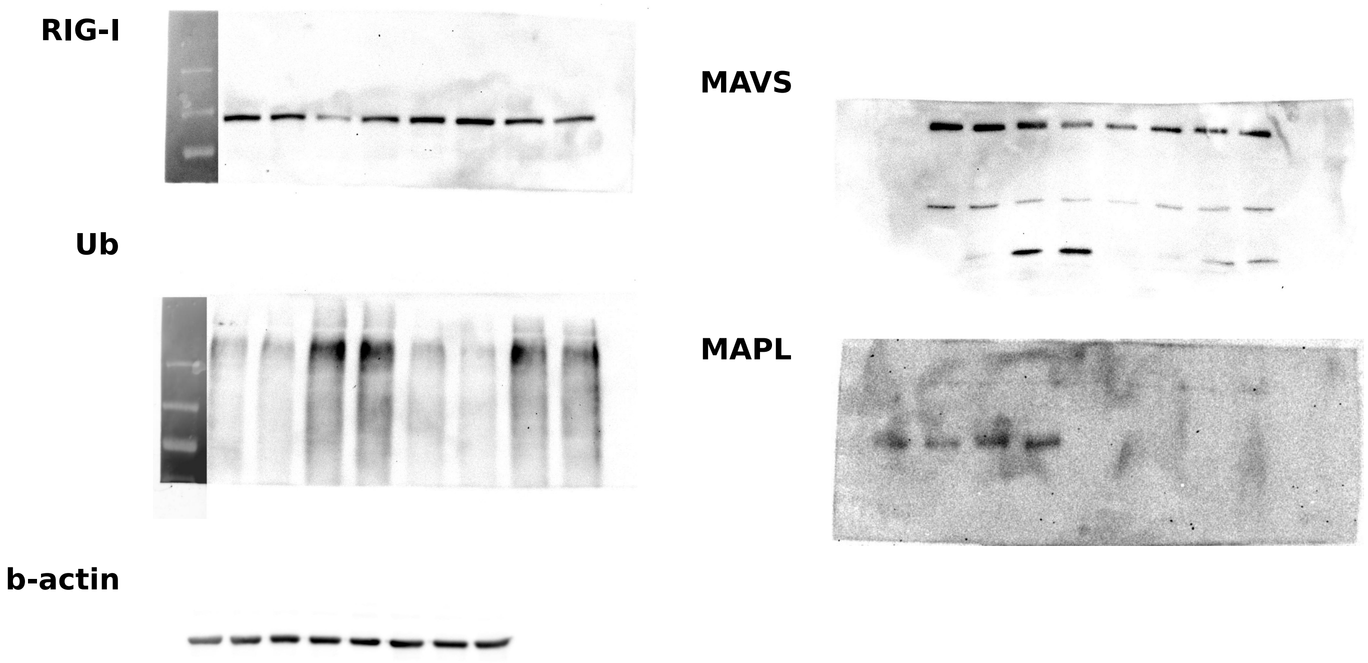

Figure 5B

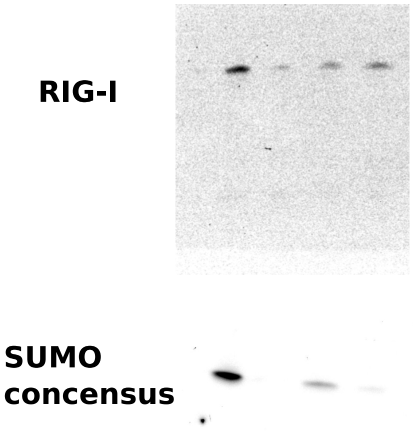

Figure 5C

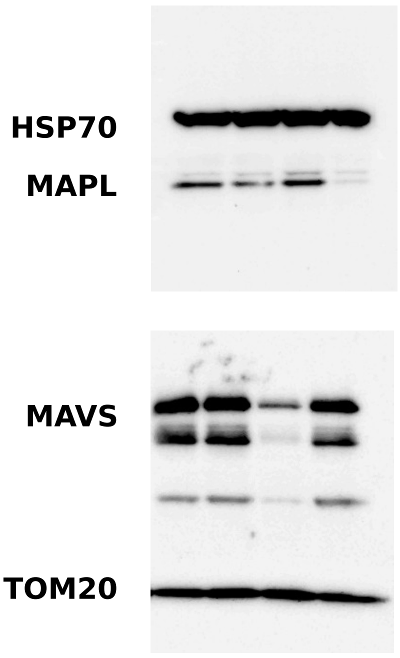

Figure S2

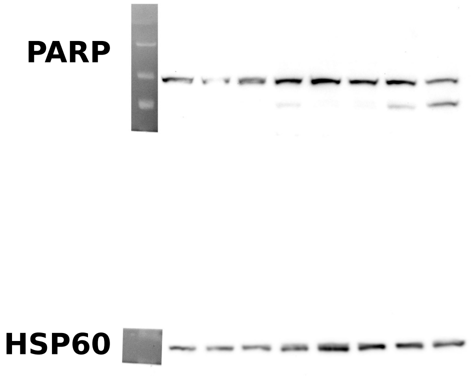

Figure S4 A

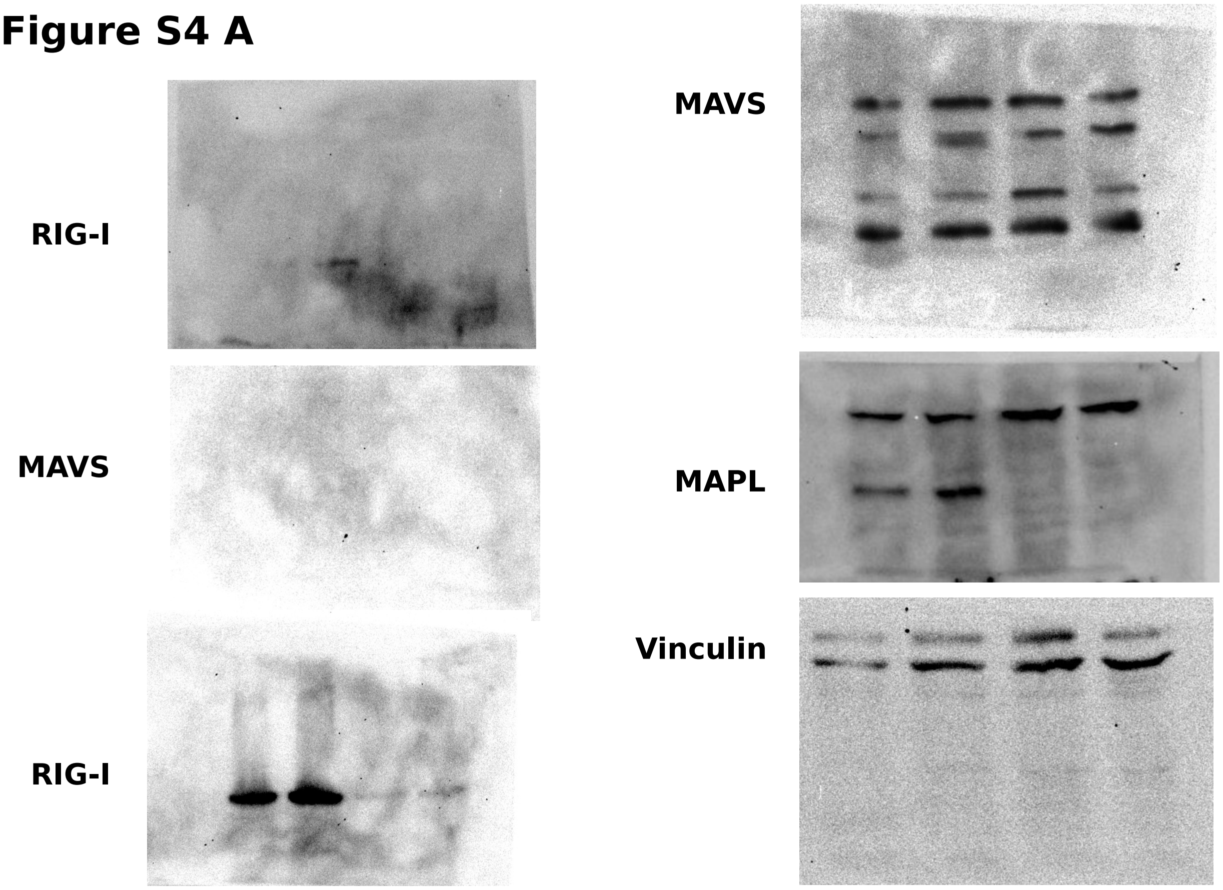

Figure S4 B

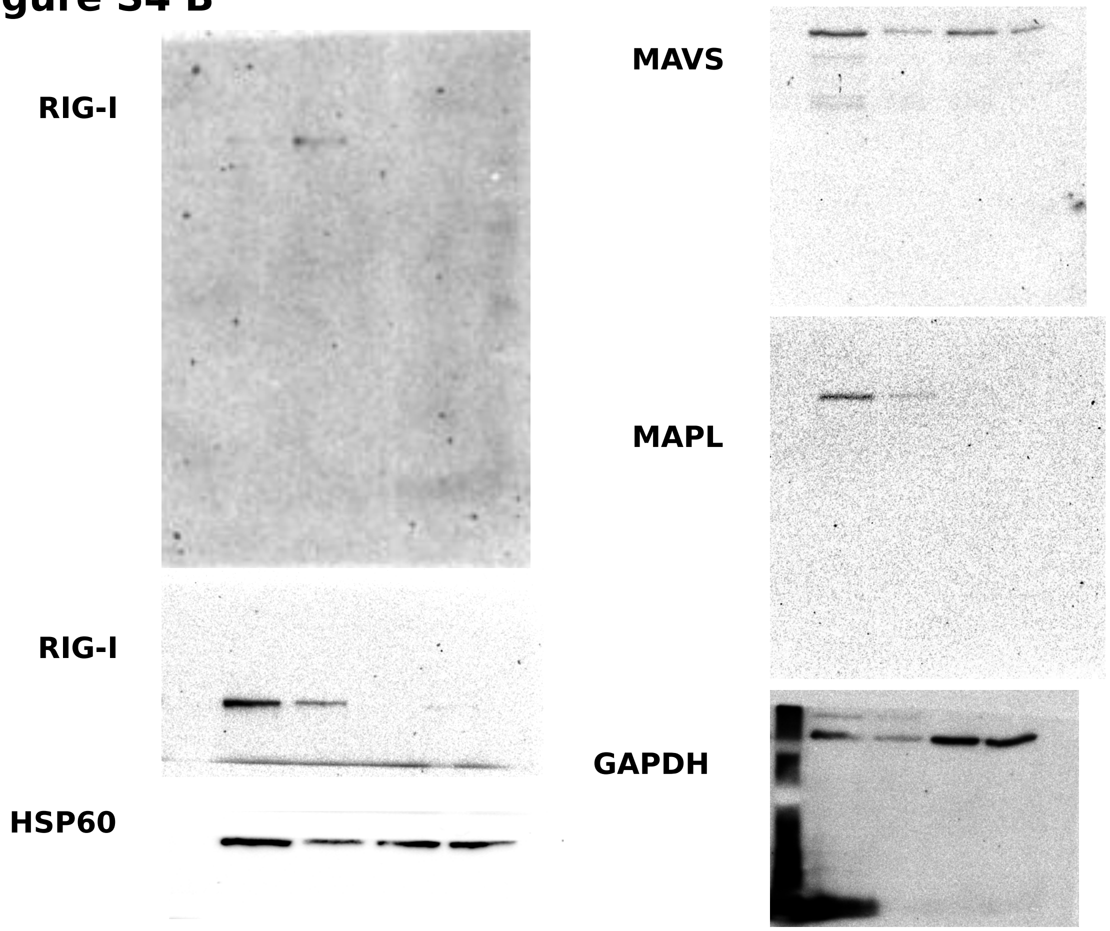

Figure S5

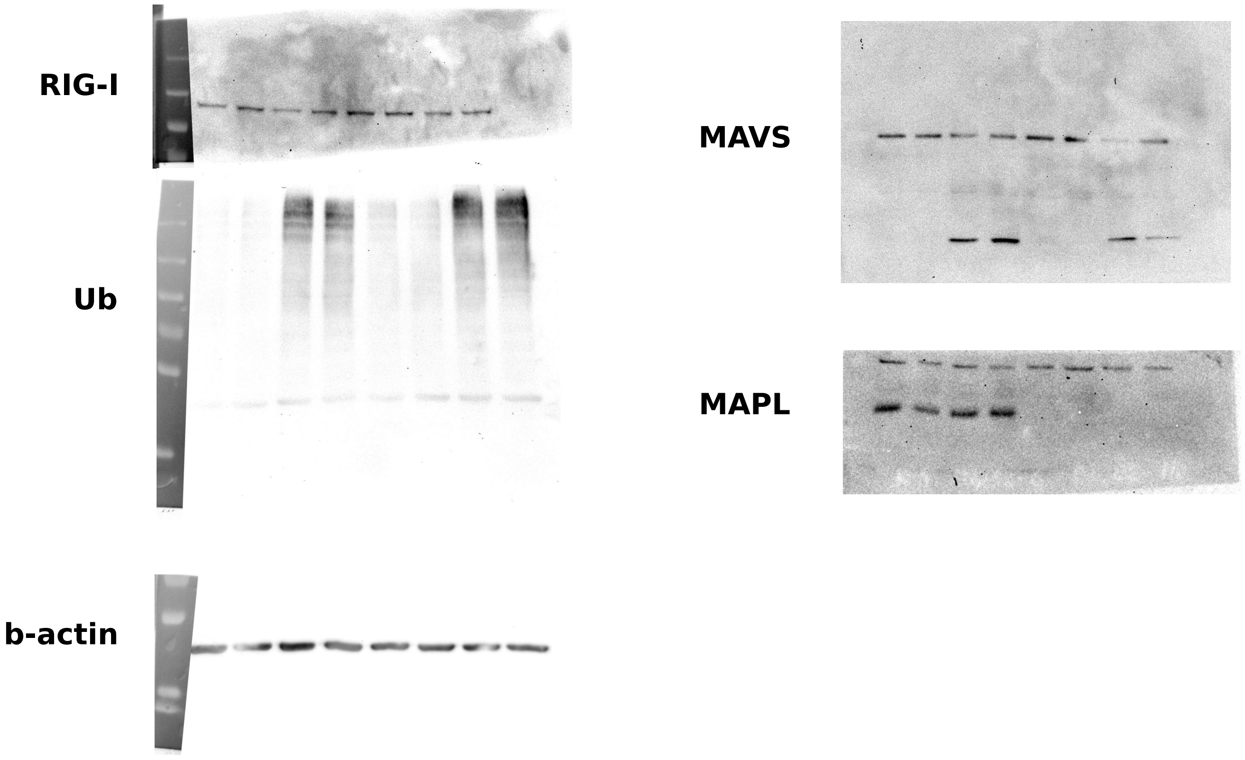

Supplement: Supplementary file 1 — Supplementary Information [file 41598_2017_151_MOESM1_ESM.pdf]
